# The Esthetic Management of Molar Incisor Hypomineralization: Succeeding the Challenge

**DOI:** 10.1155/crid/2400626

**Published:** 2025-10-13

**Authors:** Sarra Nasri, Rim Kallala, Sabra Jaâfoura, Nissaf Daouahi, Belhassen Harzallah

**Affiliations:** ^1^University of Monastir, Faculty of dental medecine of Monastir, Research Laboratory of Occlusodontics and Ceramic Prostheses, Monastir, Tunisia; ^2^Faculty of Dental Medicine, Research Laboratory of Dento-Facial, Clinical and Biological Approach (ABCDF), University of Monastir, Monastir, Tunisia

**Keywords:** ceramic veneers, dental esthetics, molar incisor hypomineralization

## Abstract

Molar incisor hypomineralization (MIH) is a developmental defect of tooth enamel that affects the quality of the enamel. It is estimated that MIH affects between 13.1% and 14.2% of the world's population. However, in some countries, such as Tunisia (North Africa), there is a dearth of information regarding the prevalence of MIH. The range of treatment options for MIH includes preventive interventions implemented during childhood as well as conservative restorations for adult patients. This case report was aimed at providing a detailed account of the esthetic rehabilitation of a clinical case involving a young female patient who had MIH-related esthetic impairment and for whom ceramic veneers were recommended as part of a minimally invasive treatment plan.

## 1. Introduction

Molar incisor hypomineralization (MIH), a developmental qualitative enamel defect, was first described by Weerheijm in 2001 [1]. The global prevalence of MIH is estimated to be between 13.1% and 14.2%, although data remain scarce in some regions, including Tunisia [2].

Clinically, MIH-affected enamel often resembles chalk or aged cheese. This porous, brittle enamel is susceptible to chipping under masticatory forces, leading to structural defects, hypersensitivity, and esthetic concerns [3]. Thus, managing MIH cases is challenging, particularly in adult patients [4].

The present paper reports a clinical case of esthetic management of MIH, respecting a therapeutic gradient approach.

## 2. Case Presentation

**Figure 1:**
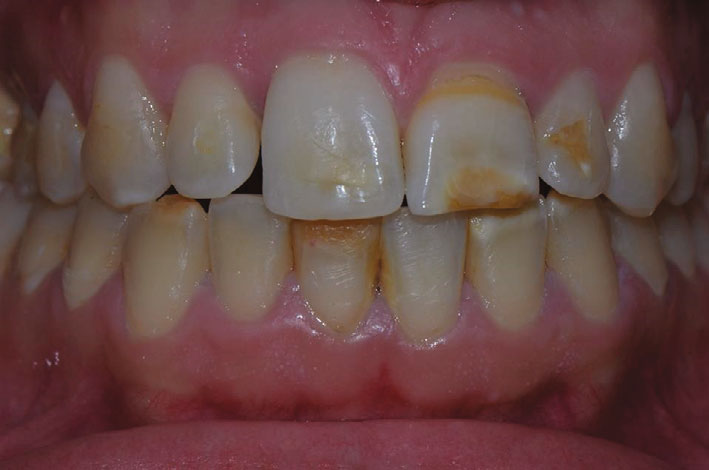
Initial situation.

A 23-year-old female patient was referred by her orthodontist to the department of prosthodontics. Her chief complaint was esthetic, related to discolorations and old composite restorations on her anterior teeth (Figure 1).

The patient's anamnesis revealed no family history of enamel abnormalities.

Intraoral examination identified extensive yellowish-white defects on the incisal edges of Teeth 11, 13, 32, 41, and 33, along with brownish defects affecting Teeth 21, 22, 41, 43, 16, 26, 46, and 36. Notably, Tooth 21 exhibited a fractured incisal edge affecting tooth morphology, while Teeth 11 and 13 had aged composite restorations (Figure 1).

Clinical assessment confirmed a diagnosis of MIH, accounting for the observed discoloration. Using the MIH-TNI index, the patient received a score of “four,” reflecting the severity and extent of the defects, as well as the presence of hypersensitivity.

The patient affirmed that she wanted a durable solution to replace unesthetic composite restorations and enhance the morphology of her upper teeth.

The esthetic management of this case required a genuine strategy to deal with the loss of substance and altered morphology associated with demarcated opacities and hypersensitivity.

Based on clinical and radiographic assessments, a treatment plan was established, involving microabrasion, in order to remove superficial enamel discolorations and minor surface irregularities. It improves appearance while preserving tooth structure. This procedure was followed by the placement of six porcelain veneers on the maxillary anterior teeth. Written informed consent was obtained from the patient, including consent for the use of clinical photographs and relevant data.

**Figure 2:**
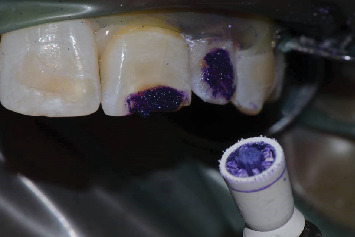
Microabrasion using Opalustre.

The initial step, microabrasion, was performed using a combination of hydrochloric acid and silicon carbide abrasive particles in a water-soluble gel (Opalustre, South Jordan, United States). This mixture was applied to the discolored enamel and gently abraded for 10 s using a contra-angle handpiece fitted with a silicon carbide abrasive point (Figure 2).

**Figure 3:**
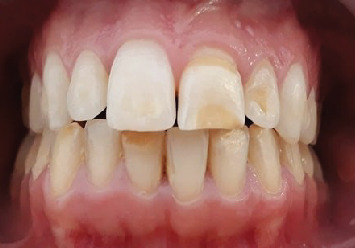
Result postabrasion.

The treatment's progress was assessed after each application on moist teeth. A noticeable reduction in brownish and yellowish discoloration was observed, which would enhance the final esthetic outcome by preventing any underlying discoloration from showing through the translucent ceramic veneers (Figure 3).

Bleaching was deliberately avoided to prevent the potential intensification of existing opaque lesions, which could have made them more noticeable. Additionally, the procedure was not performed due to the patient's reported sensitivity, as it could have further aggravated her discomfort.

**Figure 4:**
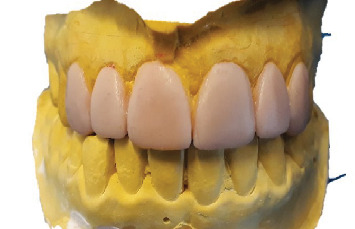
The wax-up.

**Figure 5:**
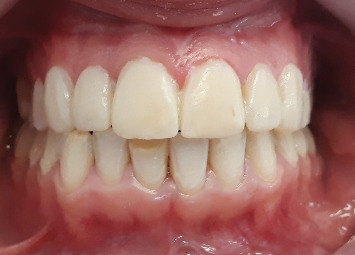
Mock-up.

After obtaining informed consent, a diagnostic wax-up was created and translated into an intraoral mock-up (Figure 4). The patient expressed satisfaction with both the esthetic and functional outcomes (Figure 5).

**Figure 6:**
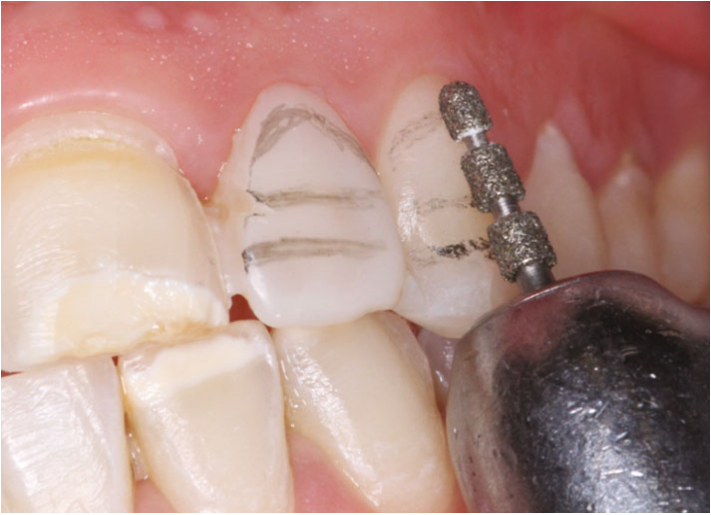
Mock-up-guided preparation using a calibrated bur.

**Figure 7:**
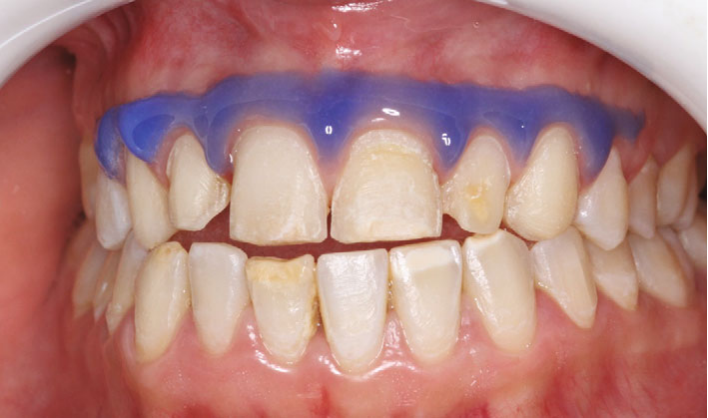
Butt margin veneer preparation on Teeth 11, 21, 12, and 23 and incisal overlap preparation on Teeth 22 and 13.

Next, a mock-up-guided preparation was carried out to ensure minimal tissue reduction, preserving as much healthy dental structure as possible (Figure 6). The finishing line on Tooth 21 was positioned slightly intrasulcularly to prevent the bonding joint from being placed on an affected area. A similar approach was applied to Tooth 11 to maintain symmetry. Preparations on Teeth 11, 21, 12, and 13 followed a butt margin design. However, the incisal preparation on Teeth 23 and 12 was displaced palatally following an incisal overlap preparation, with occlusal contacts in maximum intercuspation maintained outside of the preparation margins (Figure 7).

**Figure 8:**
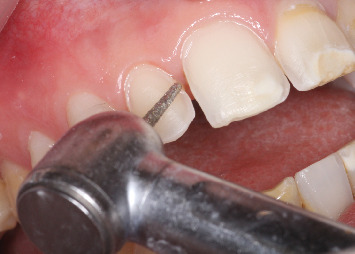
Interproximal area preparation and creation of the toboggan.

In areas where the proximal contact was maintained, the preparation was aimed at creating a toboggan underneath the contact zone (Figure 8).

A master impression was taken with polyvinyl siloxane impression material (Mono Ghenesyl, Lascod, Firenze, Italy). Shade selection was performed with the VITA 3D Master shade guide.

**Figure 9:**
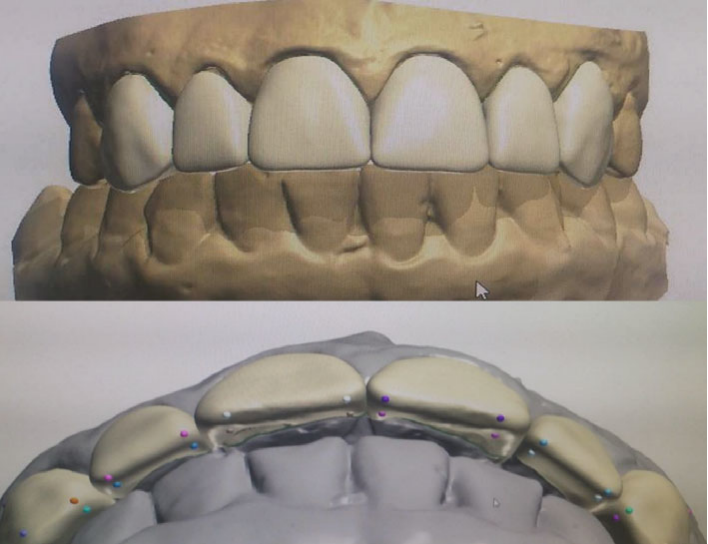
CAD/CAM conception of veneers.

**Figure 10:**
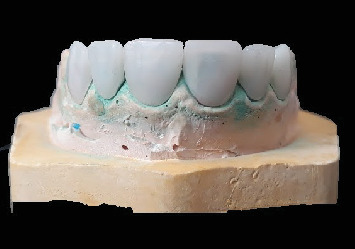
Milled ceramic veneers.

In the laboratory, the veneers were designed using “3Shape Dental System CAD” software (Figure 9), and the final restorations were fabricated from high-translucency lithium disilicate (IPS e.max CAD, Ivoclar Vivadent, shade HT A1/I12) (Figure 10).

After evaluating the morphology and marginal adaptation of the veneers on plaster cast (Figure 10), the temporary restorations were removed, and the preparations were thoroughly cleaned. Each laminate veneer was then individually assessed for proper marginal adaptation.

**Figure 11:**
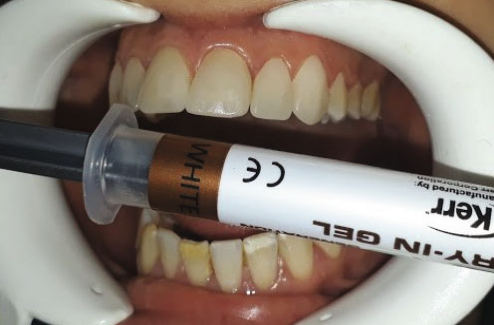
Assessing the esthetic result using the try-in gel “white.”

Using try-in pastes on each anterior quadrant, the veneers' shape, color, and overall esthetic harmony were carefully examined. To effectively mask the MIH stains, the “white” shade was selected (Figure 11).

Internal surface treatment of the ceramic veneers is a key step in the bonding procedure. Buccal surfaces of the veneers were protected by placing them on a silicon pattern, and 4.5% hydrofluoric acid solution (IPS Ceramic Etching gel, Ivoclar Vivadent) with a remarkable color was applied for 90 s and then washed under running water and air dried.

A silane-coupling agent (Monobond, Ivoclar Vivadent) was applied for 60 s according to the manufacturer's instructions.

For the dental substrate treatment, 37% orthophosphoric acid was applied for 15 s, followed by washing and air drying; the chalky aspect of teeth surfaces was noted, except for the affected areas.

Two consecutive coats of the adhesive system (Single Bond, 3M ESPE) were then applied for 15 s. It is recommended to rub it for 1 min to guarantee a deep incorporation of adhesive into tooth surface and then gently air dry for 5 s to evaporate solvents and light cure for 10 s.

The laminate veneers were luted using a light-cured luting agent. The restoration margins were light cured for 5 s on each side to facilitate the removal of excess material. Then, all restoration faces were light cured for 60 s. The maximum intercuspation occlusion was adjusted with diamond burs in areas marked with articulating paper. The laterality and protrusion of mandibular movements were checked. The adjusted surfaces were polished using polishing rubber, and the proximal surfaces finished with finishing strips (Soflex 3M ESPE).

**Figure 12:**
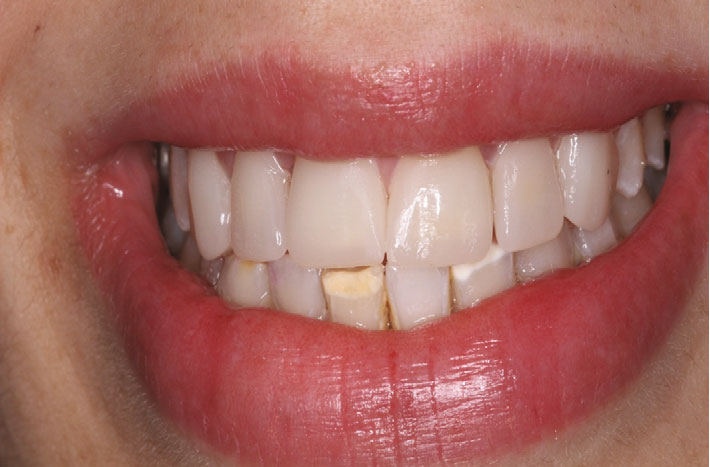
Final result.

The patient was satisfied with the immediate esthetic outcome (Figure 12).

**Figure 13:**
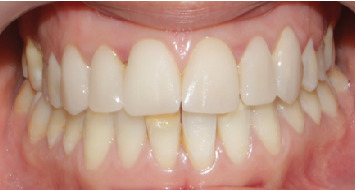
The result after 8-year follow-up.

Eight years later, she was recalled to evaluate the stability of the result (Figure 13).

The patient was demanded to respond to the Arabic version of the Psychosocial Impact of Dental Aesthetics Questionnaire (PIDAQ) to objectively assess the outcome [5].

She obtained a total score of 15 out of a possible 92, indicating a very low impact of her dentition on her psychosocial well-being. This result reflects her high satisfaction with her dental appearance and overall positive perception of her smile in social and psychological contexts.

## 3. Discussion

MIH is a worldwide recognized developmental defect affecting the enamel of permanent first molars and frequently the maxillary incisors. This condition can result in structural damage, esthetic concerns, and even functional impairment. While incisors are affected less often than molars, the severity of incisor involvement can vary considerably [3] [6].

This oral disease is a complex developmental disturbance with a multifactorial etiology. While the specific mechanisms are still being investigated, the umbrella review of Lopes et al. classified possible causes into three periods according to pre-, peri-, and postnatal periods [7].

MIH can resemble enamel hypoplasia, which represents a quantitative defect of enamel. In hypoplasia, enamel presents smooth and well-defined margins, whereas in MIH the borders of the lesion are irregular with a sharp demarcation between affected and sound enamel [8].

Clinically, MIH must also be distinguished from dental fluorosis and amelogenesis imperfecta. MIH lesions are asymmetrical and limited to certain teeth, while fluorosis affects corresponding teeth in a diffuse and bilateral pattern, often with a generalized smooth appearance. Importantly, fluorosis is associated with a history of chronic fluoride exposure during the enamel formation period, often linked to endemic regions [9, 10]. This contrasts with MIH, which occurs sporadically and does not follow a regional or familial pattern.

Regarding amelogenesis imperfecta, this hereditary condition involves all teeth with generalized and consistent defects and often presents a positive family history. In contrast, MIH presents sporadically with no familial clustering [11].

For the esthetic management of MIH and taking into consideration the stepwise therapy, several treatment options are available, varying from preventive measures to full-coverage crowns. The choice of the adequate modality must be individualized and must take into consideration the lesion severity, the color intensity, the patient's age, and expectations.

Elhannawy et al. conducted a systematic review and found that the current evidence was insufficient to make strong recommendations regarding the esthetic management of incisors affected by hypomineralization [12]. The review authors generally advocated for a conservative restorative approach that prioritizes the preservation of healthy tooth structure.

This case management followed a stepwise minimally invasive gradient. Microabrasion, involving the removal of superficial enamel (≤ 100 *μ*m) through abrasion and erosion with 18% hydrochloric or 37.5% phosphoric acid and pumice, can improve esthetics by altering the optical properties of the affected areas of enamel.

This technique is most effective for surface-level discoloration, particularly brown mottling. However, it may be unsuitable for mild MIH cases where the defect lies within the deeper enamel layers. In the presented clinical case, the microabrasion lightened the discoloration, but the esthetic improvement was insufficient, suggesting the need for additional treatment. The presence of opacities in place of the brownish stains after microabrasion suggested that tooth whitening could further aggravate these opacities. Restorative intervention was required.

Although resin infiltration is considered a conservative and esthetic treatment option, it may prove insufficient in situations where the volume and morphology of the affected teeth are compromised. In such cases, as illustrated in this report, additional restorative intervention becomes necessary to address conditions such as incisal edge fractures. Moreover, resin infiltration has some limitations regarding appearance and long-term outcomes. In cases of severe white spot lesions or deep enamel hypomineralization, the technique may not fully mask the discoloration, as the infiltrating resin primarily blends with the natural enamel but cannot completely restore areas of significant mineral loss. Moreover, the long-term durability of the treatment remains under investigation. Over time, infiltrated areas may be prone to slight discoloration or marginal changes due to wear, staining, or material degradation, which can affect the initial esthetic results and may necessitate retreatment or additional cosmetic interventions, an eventuality refused by young adult patients who are primarily seeking long-term durability [13–15].

Additive direct composite restorations had already been attempted in the anterior region but proved inadequate in achieving the patient's desired durability and esthetics, particularly in terms of long-term color stability, wear resistance, and surface gloss. In fact, several risk factors influence the longevity of composite restorations: materials, techniques, and especially patient-related factors, which play significant roles in determining outcomes [16].

Escalation to full-coverage crowns was considered disproportionate, as it would have required sacrificing a large volume of sound enamel.

Consequently, lithium disilicate ceramic veneers were selected as the next conservative step. According to the study of Poggio et al., ceramic materials demonstrated no detectable changes, confirming their suitability for use in esthetic zones, while the composite materials exhibited varying levels of surface and color alterations [17]. Moreover, thin or ultrathin ceramic veneers are effective for masking the localized hypomineralized zones [18].

On the other hand, the study of Linner et al. [19] reported the superiority of ceramic restorations (CAD/CAM ceramic) compared to resin-based restorations for MIH-affected teeth when cavity preparation is performed. The study found the highest survival rates for CAD/CAM ceramic restorations, followed by conventional composite and then noninvasive composite.

These restorations offer superior optical properties and abrasion resistance compared to composite, along with predictable adhesive bonding to enamel with minimal preparation [20]. According to the study of Panayong et al. in 2025, the survival rate of ceramic veneers was 89.3% after 10 years of follow-up [21]. However, it has been proved that this depends essentially on the preservation of enamel as the extent of dentin exposure significantly affects the survival of bonded ceramic veneers after 1–15 years of follow-up [22].

A slight reduction of 0.3–0.5 mm was performed in the cervical third to avoid overcontouring, 0.7 mm in the middle third, and 1.5–2.0 mm in the incisal third, ensuring high esthetic integration in this region. This approach allows for minimally invasive veneer preparation, preserving healthy enamel. This could be demonstrated by a whitish opaque and frosted tooth surface, once dried after orthophosphoric acid etching.

Bonding to enamel affected by MIH is more challenging than bonding to sound enamel because of its lower mineral and higher organic content. Only a limited number of studies have investigated bonding to hypomineralized enamel, largely due to difficulties in recruiting participants for in vivo studies and the scarcity of extracted MIH teeth for laboratory research. Since the excess protein in hypomineralized enamel may hinder adhesive infiltration and bonding, some authors have recommended the use of 5% sodium hypochlorite (NaOCl) as a deproteinizing agent to enhance bond strength [23].

A systematic review by Lagarde et al. [24] concluded that self-etch adhesives did not significantly improve composite bonding to MIH enamel, whereas etch-and-rinse adhesives provided better outcomes, especially when combined with deproteinization after etching, which may help preserve compromised enamel. While some studies suggest applying adhesive beforehand, others report no significant differences in shear bond strength.

For ceramic veneers, given that MIH lesions on anterior teeth are usually well demarcated and not generalized across the entire tooth surface, large areas of sound enamel remain intact, providing a reliable substrate for adhesive bonding. The preparation margin should always be placed on sound enamel by encountering and fully including the hypomineralized areas. This helps ensure better esthetic outcome and a tight bond margin.

Choosing the right shade is important to optimize the final optical properties of the tooth–veneer compound. In this case, superficial discolorations have been managed by microabrasion. However, the use of try-in pastes was of paramount help to test the masking ability of the “white” resin cement because of the translucency of lithium disilicate veneers indicated for the patient.

Although ceramic full crowns could have been considered, they were deemed too invasive for this young patient due to the tooth version and potential impact on pulp vitality. This therapeutic option could be indicated for severe cases of MIH.

## 4. Conclusion

MIH represents an enamel developmental defect, which may directly affect smile esthetics and therefore the self-esteem and personal relationship processes of an adult patient.

The treatment options, including the use of composite resin restorations or porcelain veneers, in addition to their associated advantages and limitations, should be discussed with the patient. Direct restorations may give immediate satisfaction but are not reliable as a long-term solution in contrast with ceramic veneers. As MIH-affected enamel is particularly modified and can compromise adhesion, preparation design should incorporate all affected areas. On the other hand, the bonding procedure has to be done with due care.

## Data Availability

Data sharing is not applicable to this article as no datasets were generated or analyzed during the current study.
